# m5C: Novel Diagnostic and Drug Repurposing Targets for Nonalcoholic Steatohepatitis

**DOI:** 10.1155/ijog/4309290

**Published:** 2026-02-25

**Authors:** Shuxian Chen, Renquan Duan, Jingyi Qiu, Zhiyu Lei, Wei Chen, Xiumei Li

**Affiliations:** ^1^ Department of Gastroenterology, Xiangyang No.1 People′s Hospital, Hubei University of Medicine, Xiangyang, China, hbmu.edu.cn; ^2^ Department of General Surgery, Zaoyang No.1 People′s Hospital, Zaoyang, China; ^3^ The Fourth Clinical College of Hubei University of Medicine, Shiyan, China

**Keywords:** drug repurposing, m5C, machine learning, multiomics, nonalcoholic steatohepatitis

## Abstract

**Background:**

Epigenetic medication, such as RNA 5‐methylcytosine (m5C), is well‐recognized as a key regulator in hepatic metabolism and immune responses. However, m5C regulatory mechanisms in NASH pathogenesis have not yet been clearly elucidated.

**Methods:**

By utilizing three bulk profiles of NASH patients acquired from GEO and integrative bioinformatic pipelines, such as Limma framework, consensus clustering, and machine learning, we first identified m5C‐related molecular subgroups and hub genes for NASH patients. Besides, diagnostic performance and biological characteristics of m5C‐related hub gene were estimated at bulk level. Indeed, the heterogeneity of m5C‐related hub gene for NASH patients was deciphered in single‐cell transcriptomic profiles at temporal and spatial manners, especially in artificial intelligence (AI)‐driven virtual cells. Furthermore, potential therapeutic agents targeting m5C‐associated hub genes for the treatment of NASH were enriched by AI‐driven drug enrichment framework (DrugReflector) based on NASH bulk profile and then validated by molecular docking. Finally, in vitro studies quantified the expression of m5C‐associated hub genes compared to normal control.

**Results:**

m5C can divide NASH patients into two various consensus groups with different molecular and immune patterns. Furthermore, ERCC2 and FOXC2 can be considered two upregulated m5C‐associated hub genes involved in NASH pathogenesis, which were mainly distributed at cholangiocyte. BRD‐K93672499 can be considered a multitarget therapeutic strategy targeting ERCC2 and FOXC2 for the treatment of NASH.

**Conclusion:**

Our study first deciphered the m5C in predictive and therapeutic potential for NASH patients, which gains more insight into their personalized and precision medicine.

## 1. Introduction

Nonalcoholic steatohepatitis (NASH), an advanced subtype of nonalcoholic fatty liver disease (NAFLD), manifests with hepatic fat accumulation, inflammatory infiltration, and fibrotic remodeling, which may ultimately progress to cirrhosis or liver cancer [1]. As obesity and metabolic syndrome continue to rise worldwide, NASH has emerged as a leading contributor to chronic liver disorders and an escalating challenge to global health [2]. Despite notable advances in elucidating its underlying metabolic pathways and immunological processes, effective pharmacological treatments remain lacking [3]. Increasing evidence indicates that NASH pathogenesis involves intricate interactions among lipid metabolic disturbances, oxidative stress, immune dysregulation, and epigenetic alterations [4]. Within the realm of epigenetic regulation, RNA 5‐methylcytosine (m5C) serves as an evolutionarily conserved modification occurring at the posttranscriptional level [5]. It plays crucial roles in RNA stability, transport, and translation regulation, primarily catalyzed by NSUN family methyltransferases (NSUN1–7) and DNMT2 and dynamically oxidized by TET enzymes [6]. Functionally, m5C affects RNA structure and protein–RNA interactions, while reader proteins such as ALYREF and YBX1 mediate m5C‐dependent RNA export and expression [6]. Recent studies have shown that m5C dysregulation contributes to tumorigenesis and metabolic disorders [7]. For example, NSUN2‐mediated m5C stabilizes MYC mRNA to promote hepatocellular carcinoma proliferation, while aberrant m5C influences metabolic reprogramming and drug resistance in colorectal cancer [6, 7]. Moreover, m5C participates in oxidative stress, mitochondrial dysfunction, and immune activation key processes in liver disease progression [8]. Aberrant m5C modification may regulate lipid metabolism, inflammatory signaling, and hepatocyte apoptosis, exacerbating lipotoxic injury and immune remodeling [8]. Despite advancement of m5C into hepatic issues, there are still no more insights into the m5C in the NAFLD and NASH pathogenesis.

**Figure 1:**
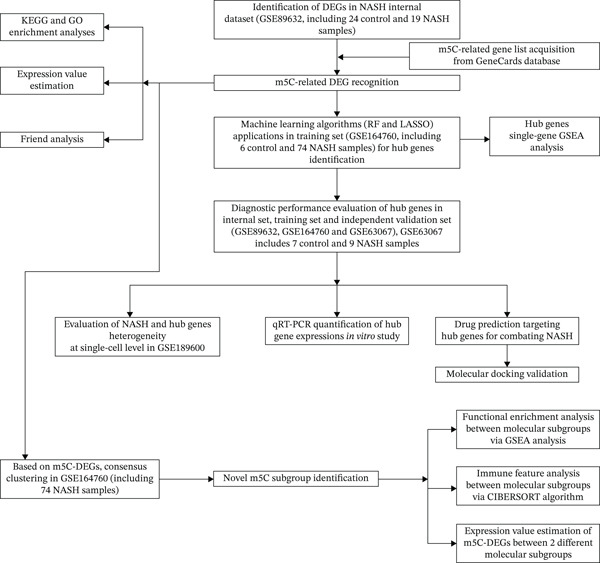
The workflow of this study.

Here, we integrated multiomics data with artificial intelligence (AI) pipelines, such as machine learning and deep learning for elaborating the predicative and therapeutic potentials of m5C for NASH patients. Results showed that m5C can stratify NASH into two biological different molecular subgroups and discovered that ERCC2 and FOXC2 can be considered two upregulated m5C‐associated predictive and therapeutic targets involved in NASH pathogenesis, which were mainly distributed at cholangiocyte. Besides, we also identified BRD‐K93672499 can be considered an optimal therapeutic framework targeting ERCC2 and FOXC2 for the treatment of NASH. The overall analytical framework of this research is illustrated in Figure 1.

## 2. Methods

### 2.1. Source of Data

The bulk profile of GSE89632, GSE164760, and GSE63067 was obtained from the Gene Expression Omnibus (GEO) database. GSE89632, GSE164760, and GSE63067 were designated as internal, training, and independent validation sets, respectively. We downloaded all datasets via the GEOquery package of R [9]. For datasets in which raw expression values had not undergone prior standardization, quantile normalization was implemented through the Limma package in R [10].

### 2.2. Identification of DEGs

Differential expression analysis of the GSE89632 dataset was carried out in R with the limma package, setting the criteria at an adjusted *p* value below 0.05 and |log_2_FC| exceeding 0.5 [10]. The resulting DEGs were then overlapped with the m5C gene list to identify m5C‐associated DEGs, which were displayed using Venn diagrams. Ultimately, the ggplot2 package in R was utilized to construct volcano and heatmap visualizations, depicting the expression patterns of the identified DEGs. FRIEND analysis was conducted with the GOSemSim algorithm to rank the importance of m5C‐associated DEGs. The chromosomal localization of m5C‐associated DEGs was determined using the human genome reference via the circlize package of R. Subsequently, GO and KEGG enrichment analyses were carried out to uncover major signaling cascades and metabolic processes involved via the clusterProfiler package of R.

### 2.3. Consensus Clustering

The ConsensusClusterPlus package in R was applied to the GSE164760 cohort to classify NASH patients into distinct molecular subtypes based on m5C‐associated DEGs [11]. The most appropriate clustering partition number was determined by calculating the relative change in the area under the cumulative distribution function (CDF) curve across a range of cluster values. The clustering algorithm was repeated multiple times, and the final partitioning identified two distinct subgroups, C1 and C2 [11]. Immune infiltration analysis was performed between C1 and C2 via the CIBERSORT algorithm of R [12]. Additionally, molecular expression analysis identified m5C‐related DEGs between the subgroups, further elucidating the biological heterogeneity between molecular subgroups via gene set enrichment analysis (GSEA) in accordance with the hallmark gene set downloaded from the MSIGDB database via the clusterProfiler package of R [13].

### 2.4. Machine Learning Algorithms

In this study, we applied random forest (RF) and LASSO machine learning algorithms to identify key biomarkers for NASH in GSE164760. LASSO logistic regression was first employed to select the most important features in GSE164760, with the optimal regularization parameter (*λ*) chosen via cross‐validation [14]. The efficiency of the LASSO regression model was assessed through binomial deviance analysis to identify the genes with the greatest predictive impact. Next, we used the RF algorithm to rank the features by their importance, assessing their significance through mean decrease accuracy and Gini index in GSE164760 [15]. After intersecting the LASSO and RF results, we acquired m5C‐associated hub genes. Single‐gene GSEA was then conducted to analyze the biological pathways associated with the identified genes in accordance with the hallmark gene set downloaded from the MSIGDB database via the clusterProfiler package of R [13]. We also estimated the coexpression patterns of m5C‐associated hub genes with m5C key modulators and then visualized by ggplot2 package of R.

### 2.5. Diagnostic Performance Evaluation

We developed a predictive model based on the datasets GSE89632, GSE164760, and GSE63067 through receiver operating characteristic (ROC) and precision–recall (PR) curves generated by the pROC and rms packages in R. The model reliability was further substantiated through nomogram and calibration with DCA using the rms and ResourceSelection packages, which compared the predicted probabilities with the actual clinical outcomes [16].

### 2.6. Single‐Cell Analysis

To commence our study, we acquired the single‐cell transcriptomic dataset of NASH (GSE189600) from the GEO database. The analysis of the single‐cell RNA sequencing (scRNA‐seq) data encompassed several crucial procedures, including quality control (QC), dimensionality reduction, and marker identification, all executed utilizing the Seurat R package [17]. We implemented QC measures on each individual cell, adhering to specific criteria that required gene counts to range between 200 and 6000, a unique molecular identifier (UMI) count to exceed 1000, and a mitochondrial gene percentage to remain below 10%. Following the QC procedures, the data underwent normalization, which facilitated the identification of 2000 genes exhibiting notable variability for further analysis. Postnormalization, we applied dimensionality reduction methods, specifically *t*‐SNE and UMAP. Cell type annotations were performed using the scMayoMap algorithm within the R environment [18]. Intercellular communication networks were inferred utilizing the Celltalk package in R [19]. Furthermore, the exploration of energy metabolic pathways at the single‐cell level among the annotated cell populations was carried out through the scMetabolism package in R [20]. Additionally, pseudotime analysis of targeted gene expression within specific cell types was executed using the monocle2 package in R [21]. ScTenifoldKnk was performed for identification of knockout (KO) of hub gene in targeted cell [22].

### 2.7. Cell Lines and Culture

After thawing and recovery, LO‐2 cells were maintained in DMEM/F12 supplemented with 10% FBS and incubated at 37°C under a humidified atmosphere containing 5% CO_2_. When the cells proliferated to 75%–85% confluence, they were digested, passaged, and cryopreserved. The fatty acid mother solution was prepared by mixing sodium oleate and sodium palmitate at a molar ratio of 2:1. The fatty acid mother solution was then diluted to 1.2 mmol/L to create the fatty acid induction solution. LO‐2 cells were divided into a control group and a NASH model group. The control group cells were cultured in complete medium, while the model group cells were cultured in the 1.2 mmol/L fatty acid induction solution. After 24 h of intervention, the relevant indicators of the cells in each group were measured.

### 2.8. RNA Extraction and qRT‐PCR Quantification

Total RNA was extracted using TRIzol reagent (TaKaRa, Beijing, China), and subsequent assessments of its concentration, purity, and integrity were performed with a NanoDrop spectrophotometer (Thermo Scientific, Waltham, MA, United States). For the reverse transcription process, 1 *μ*g of total RNA was employed in conjunction with HiScript II Q RT SuperMix for quantitative PCR (qPCR), which includes both a gDNA wiper and a gDNA eraser (Vazyme, Shanghai, China). The concentration, purity, and integrity of the synthesized complementary DNA (cDNA) were evaluated using the aforementioned NanoDrop spectrophotometer. The quantitative reverse transcription polymerase chain reaction (qRT‐PCR) was executed utilizing SYBR Green MasterMix (11203ES50, YEASEN, Shanghai, China) and StepOne Software Version 2.3 (Applied Biosystems, Carlsbad, CA, United States) over 40 cycles, with three biological replicates for each sample. Data analysis was performed using the ΔΔCt (cycle threshold) method, with expression levels normalized to the reference gene, GAPDH. The primer sequences used in the qRT‐PCR assays are detailed below:

*ERCC2:*

F: 5 ^′^‐CTGGAGGTGACCAAACTCATCTA‐3 ^′^

R: 5 ^′^‐CCTGCTTCTCATAGAAGTTGAGC‐3 ^′^

*FOXC2:*

F:5 ^′^‐GCCTAAGGACCTGGTGAAGC‐3 ^′^

R:5 ^′^‐TTGACGAAGCACTCGTTGAG‐3 ^′^

*GAPDH:*

F:5 ^′^‐GAGAAGGCTGGGGCTCATTT‐3 ^′^

R:5 ^′^‐ATGACGAACATGGGGGCATC‐3 ^′^

### 2.9. Western Blotting

After the administration of various treatments, the cells were thoroughly washed with ice‐cold phosphate‐buffered saline (PBS) (Hyclone, Seattle, WA, United States) and subsequently collected via gentle scraping. Total protein extraction was accomplished by lysing the cells with radioimmunoprecipitation assay (RIPA) lysis buffer (Beyotime, Shanghai, China), supplemented with a combination of phosphatase inhibitors (Beyotime, China) and protease inhibitors (Beyotime, China). The resulting cell lysates were centrifuged at 14,000 × *g* for 15 min at 4°C. Postcentrifugation, the lysates were denatured for 10 min in a 5× SDS‐PAGE loading buffer (Beyotime, China). The proteins were then separated using SDS‐PAGE and subsequently transferred to polyvinylidene fluoride (PVDF) membranes (Beyotime, China) for Western blot analysis. The membranes were blocked with NcmBlot blocking buffer (NCM Biotech, Suzhou, China) for 10 min. Following this, they were incubated with primary antibodies for 8 h at 4°C, diluted in 5% bovine serum albumin (BSA) (Solarbio, Beijing, China). After the incubation, the membranes were exposed to secondary antibodies (ThermoFisher, Waltham, MA, United States), diluted in WB secondary antibody diluent solution (Beyotime, Shanghai, China) at a 1:1000 dilution for 2 h at room temperature. Protein detection was carried out using an enhanced chemiluminescence (ECL) substrate (Thermo Fisher, Waltham, MA, United States). The quantification of protein expression was performed by analyzing the band densities of the target proteins using ImageJ software Version 1.57, based on density values relative to the GAPDH protein. The primary antibodies employed in this investigation included FOXC2 (ab5060, ABCAM, United States: 1:1000), ERCC2 (ab167418, ABCAM, United States: 1:1000), and GAPDH (ab181602, ABCAM, United States: 1:2000).

### 2.10. Drug Prediction and Molecular Docking

DrugReflector represents an active learning framework that utilizes transcriptomic data to identify modulators associated with disease phenotypes. Utilizing the dataset GSE164760, we implemented DrugReflector to determine the most effective therapeutic agents aimed at mitigating NASH [23]. To evaluate the binding affinity of the optimal therapeutic agents with the m5C‐associated hub genes, molecular docking studies were conducted. This technique was employed to investigate the interactions between the selected drugs and their corresponding protein targets. The Protein Data Bank (PDB) files for the target proteins were obtained from the RCSB PDB, while ligand SDF files were sourced from the PubChem database. Following this, molecular docking was performed to estimate the binding affinities between the targeted proteins and the compounds of interest. Initially, PyMOL software (Version 2.6.0) was utilized to remove water molecules and ligands, thereby retaining only the protein backbone. Subsequently, the AutoDock Vina Tool (Version 4.2.6) was employed to identify potential binding sites on the protein surface and to conduct flexible molecular docking studies. This process involved the calculation of docking scores and binding affinities (expressed as Vina scores in kilocalorie per mole) for each identified binding site, ranking the top five based on their binding energies, and ultimately selecting the binding site with the lowest energy for visualization purposes in PyMOL. The resultant images illustrated the positions of hydrogen bonds related to ligand binding [24]. Finally, the results were depicted in PyMOL to showcase the binding modes and hydrogen bonding interactions effectively.

### 2.11. Statistical Analysis

All statistical procedures were performed using R software. Data normality was first assessed, and datasets following a Gaussian distribution were analyzed with a paired *t*‐test, while those deviating from normality were compared using the Wilcoxon rank‐sum test. A two‐tailed *p* value less than 0.05 was deemed statistically significant. Spearman correlation analysis was conducted to determine gene coexpression patterns and explore relationships between gene expression levels and immune cell infiltration.

## 3. Results

### 3.1. Identification of m5C‐Associated DEGs for NASH Patients

First, the GSE89632 dataset obtained from the GEO database was processed to remove batch effects and perform normalization (Figure 2a,b). Subsequently, DEGs in GSE89632 were identified through standard procedures (Figure 2c). A cross‐analysis between the m5C gene list and DEGs led to the identification of 10 m5C‐associated DEGs, and their similar importance with expression patterns and chromosomal distribution was also examined (Figures 2d, 2e, 2f, and 2g). Additionally, the functional roles and potential mechanisms of these 10 m5C‐associated DEGs were assessed using KEGG and GO analyses (Figure 2h,i).

Figure 2Identifcation of m5C‐associated DEGs for NASH patients. (a) PCA plot of GSE89632. (b) Box plot of GSE89632. (c) Volcano plot of DEGs in GSE89632. (d) Venn diagram of m5C‐associated DEGs. (e) Circular plot showing the chromosomal distribution of m5C‐associated DEGs. (f) Expression levels of m5C‐associated genes. (g) Heatmap of m5C‐associated DEGs. (h) GO enrichment analysis of m5C‐associated DEGs. (i) KEGG enrichment analysis of m5C‐associated DEGs.

**Figure:**
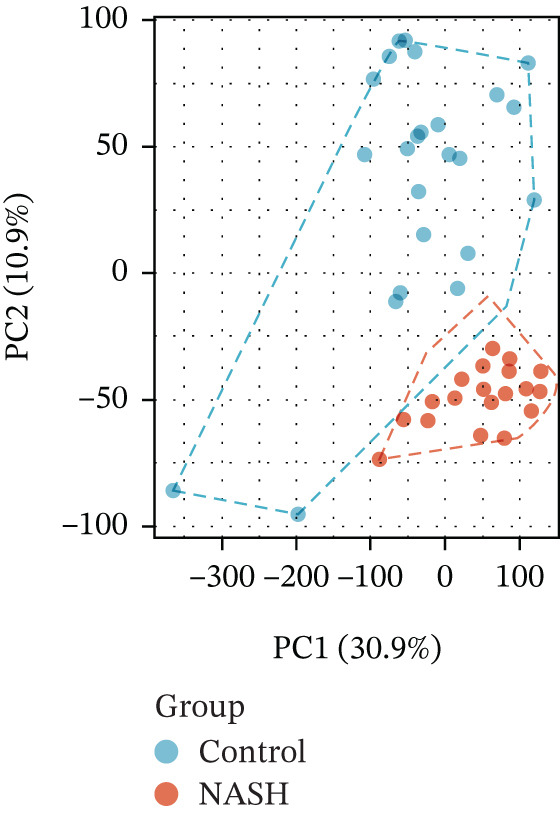
(a)

**Figure:**
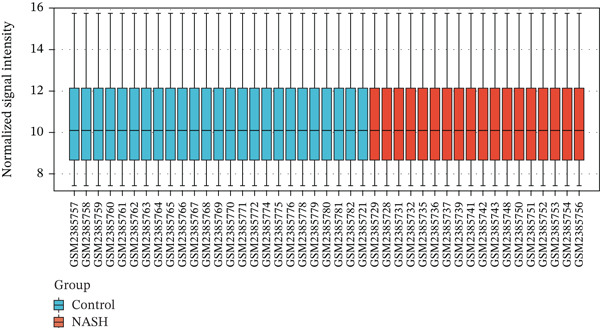
(b)

**Figure:**
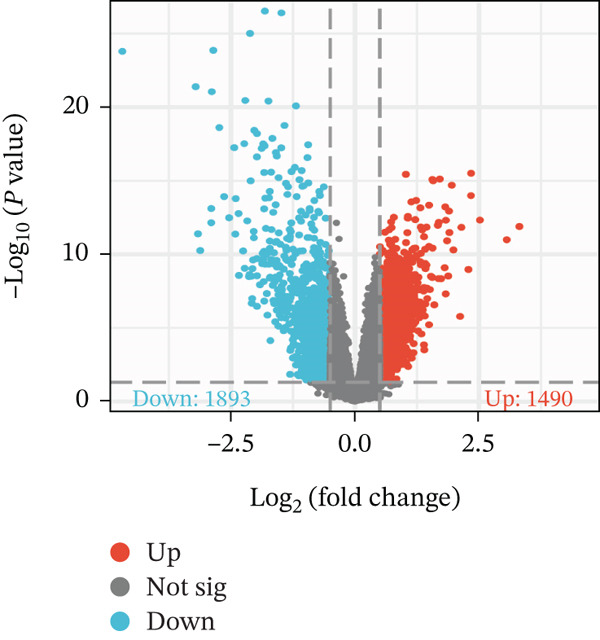
(c)

**Figure:**
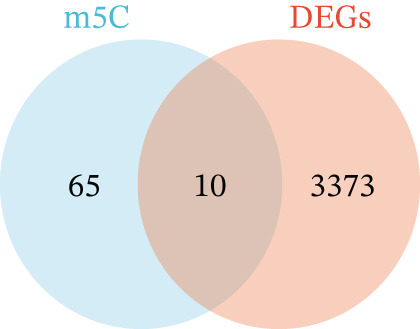
(d)

**Figure:**
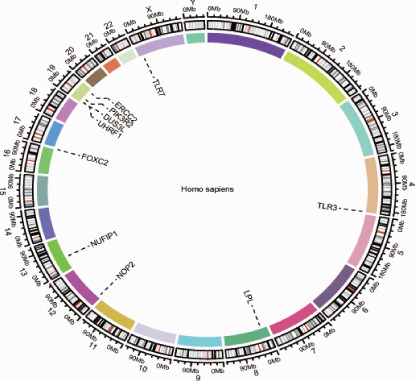
(e)

**Figure:**
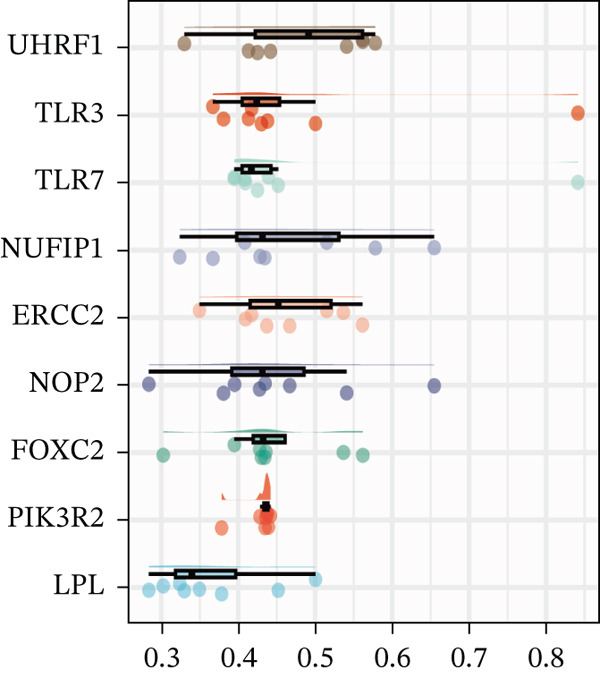
(f)

**Figure:**
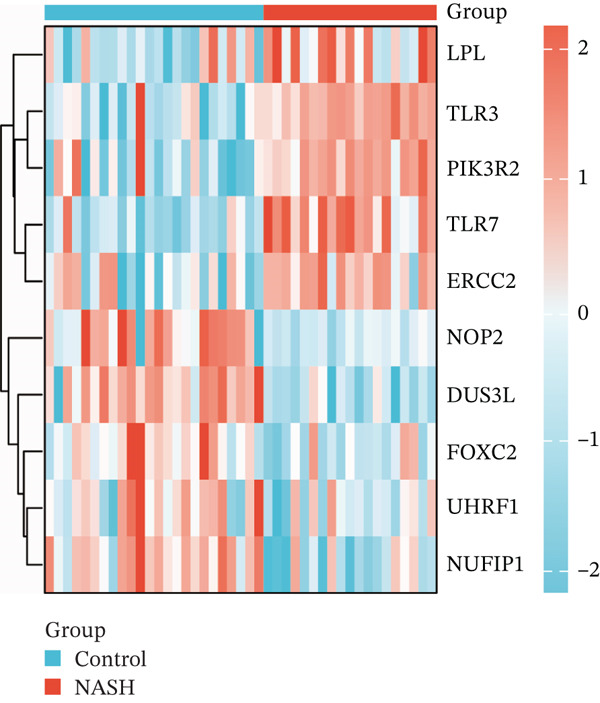
(g)

**Figure:**
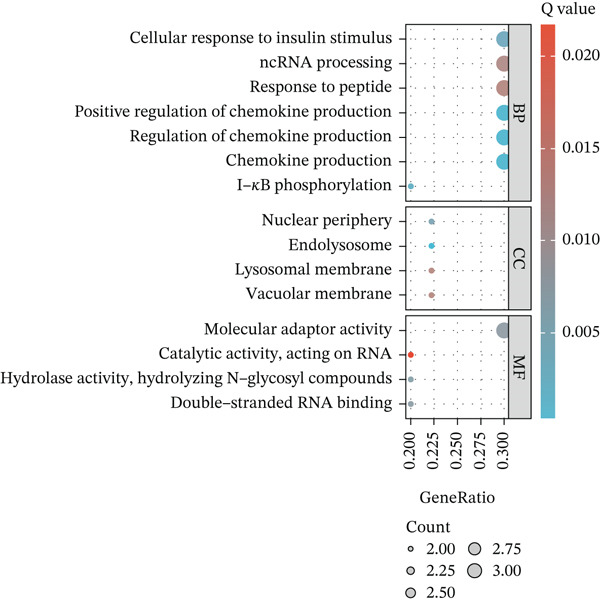
(h)

**Figure:**
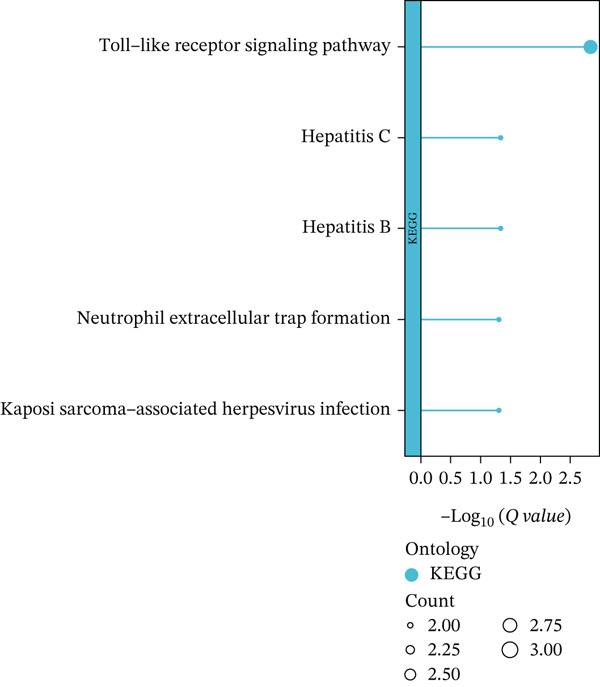
(i)

### 3.2. m5C‐Related Subgroup Identification for NASH Patients via Consensus Clustering

Subsequently, we identified m5C‐related subgroups in NASH patients using consensus clustering. First, we classified the m5C subtypes based on m5C‐associated DEGs obtained from the GSE164760 dataset. The results indicated that m5C‐associated DEGs were able to divide NASH patients into two molecular subgroups, named C1 and C2 (Figures 3a, 3b, and 3c). Additionally, we identified the expression differences of DEGs between the C1 and C2 subgroups, along with their functional discrepancies (Figure 3d,e). Furthermore, analysis of immune infiltration demonstrated notable variations in immune activity between the C1 and C2 molecular subgroups (Figure 3f).

Figure 3Identifcation of m5C‐associated molecular subgroups for NASH patients. (a) Consensus CDF plot of consensus clustering result. (b) Delta area plot of consensus clustering result. (c) PCA plot of C1 and C2. (d) GSEA ridge plot illustration between C1 and C2. (e) Heatmap illustration of DEGs in C1 and C2 subgroups. (f) Immune infiltration analysis between C1 and C2.

**Figure:**
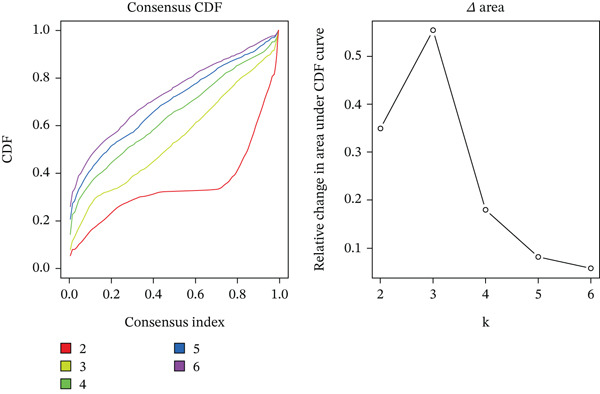
(a)

**Figure:**
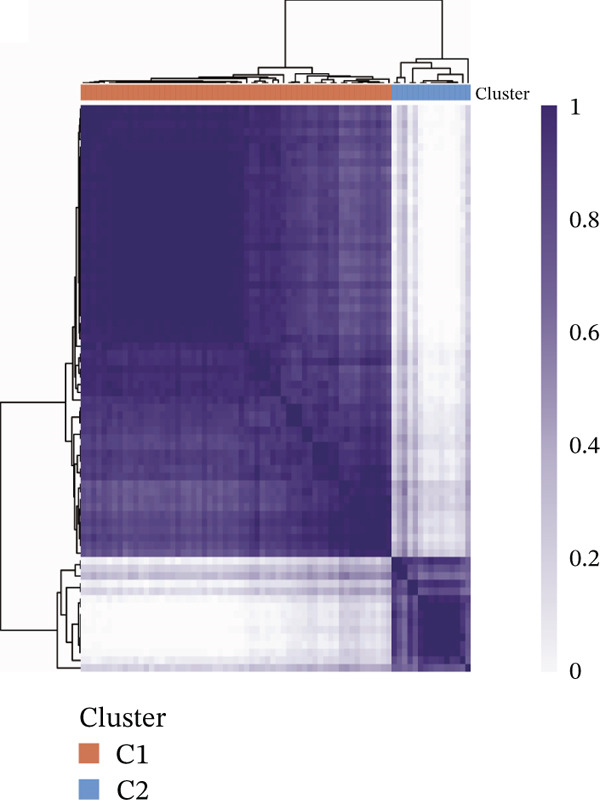
(b)

**Figure:**
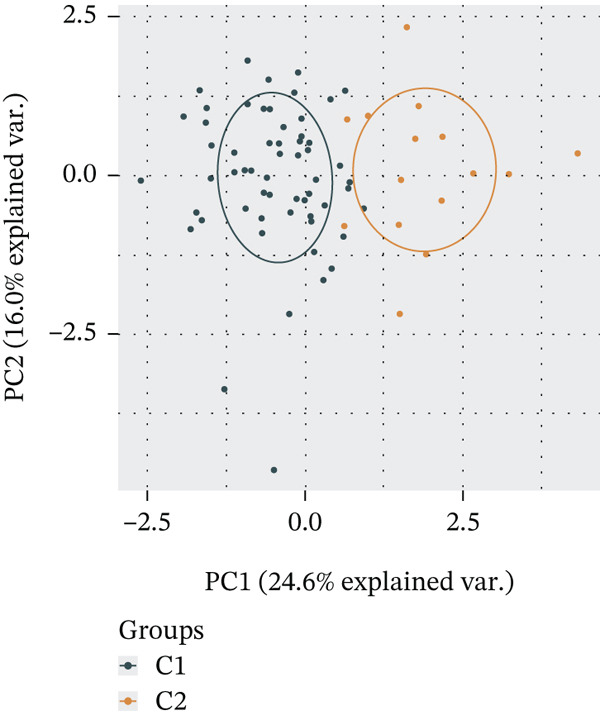
(c)

**Figure:**
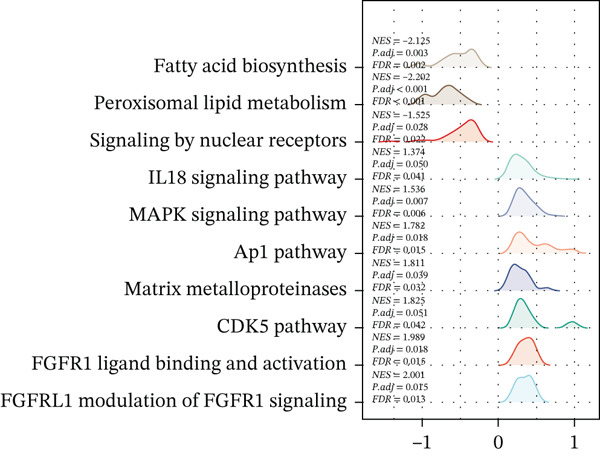
(d)

**Figure:**
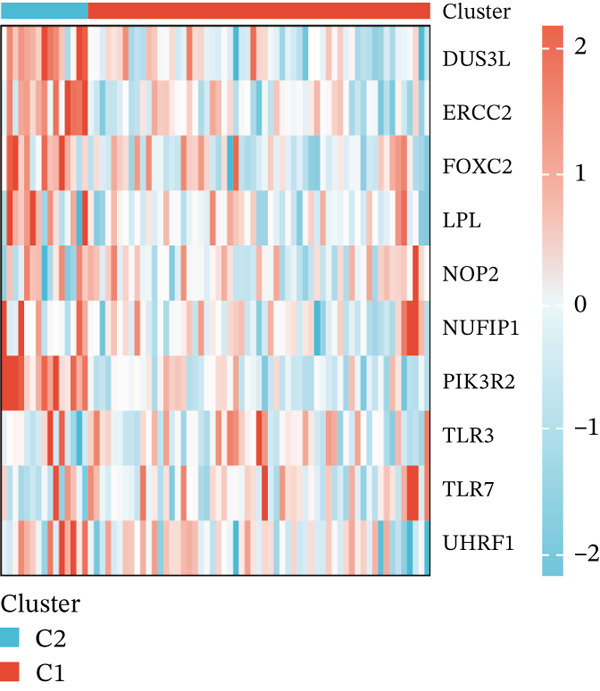
(e)

**Figure:**
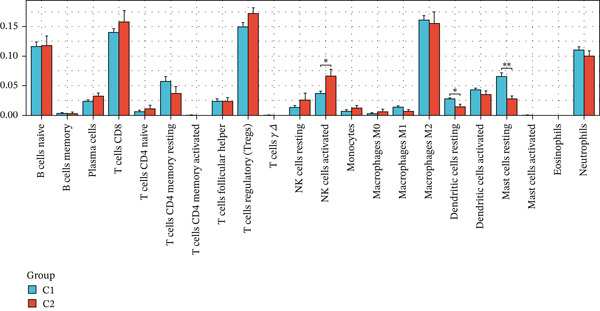
(f)

### 3.3. m5C‐Related Hub Variable Identification for NASH Patients

After extracting samples from the TCGA‐NASH cohort and obtaining corresponding bulk sequencing and clinical data, we performed LASSO‐Cox regression using RF analysis (Figure 4a,b). Subsequently, two key hub genes, FOXC2 and ERCC2, were identified (Figure 4c). GSEA additionally uncovered strong correlations between these genes and key biological processes, including glycolytic metabolism and the unfolded protein response (Figure 4d,e). Besides, we also estimated the coexpression patterns between FOXC2 and ERCC2 and key m5C regulators (Figure 4f,g). Results have shown that FOXC2 was coexpressed with FMR1, and ERCC2 was coexpressed with YBX1, TET1, and FMR1 (Figure 4f,g).

Figure 4m5C‐associated hub gene identification for NASH patients. (a) LASSO regression results. (b) Random forest results. (c) Intersection of LASSO and RF results. (d) GSEA of FOXC2 in NASH. (e) GSEA of ERCC2 in NASH. (f) Coexpression heatmap between FOXC2 and m5C regulators. (g) Coexpression heatmap between ERCC2 and m5C regulators.

**Figure:**
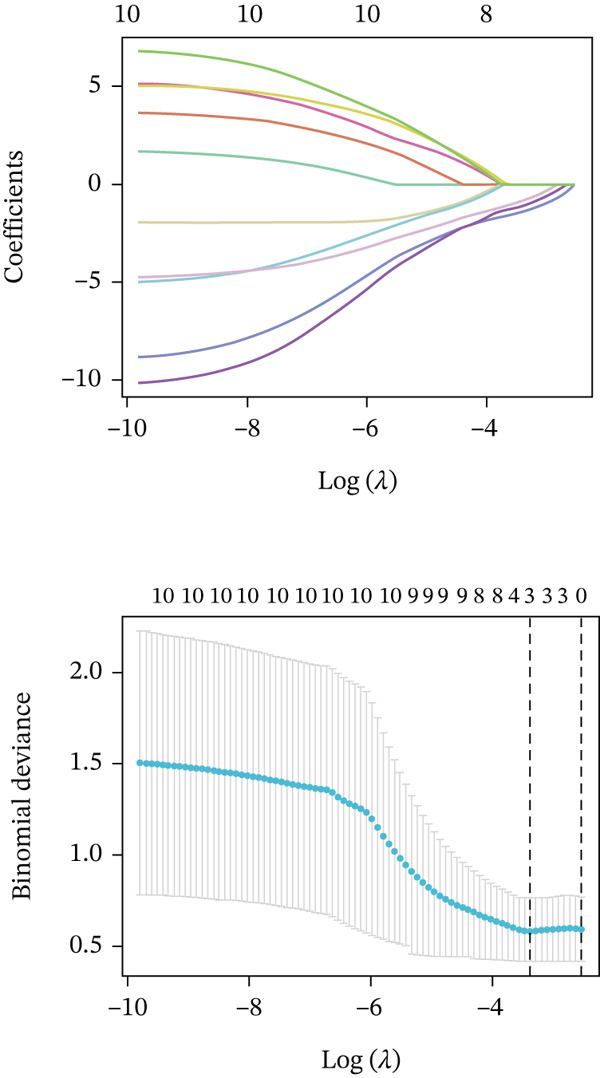
(a)

**Figure:**
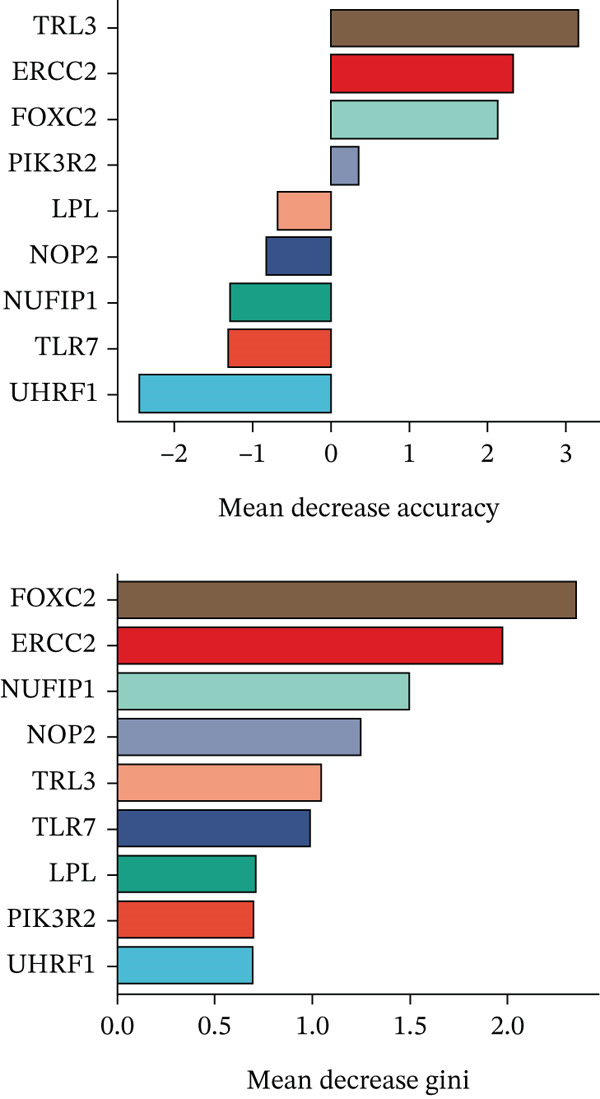
(b)

**Figure:**
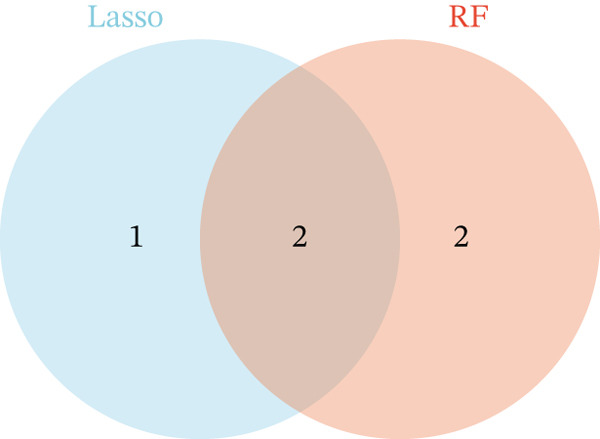
(c)

**Figure:**
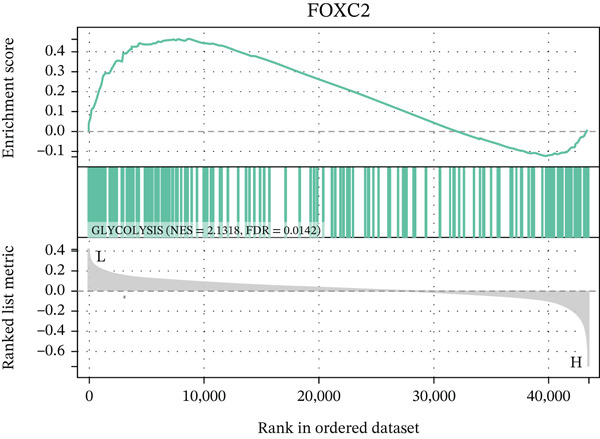
(d)

**Figure:**
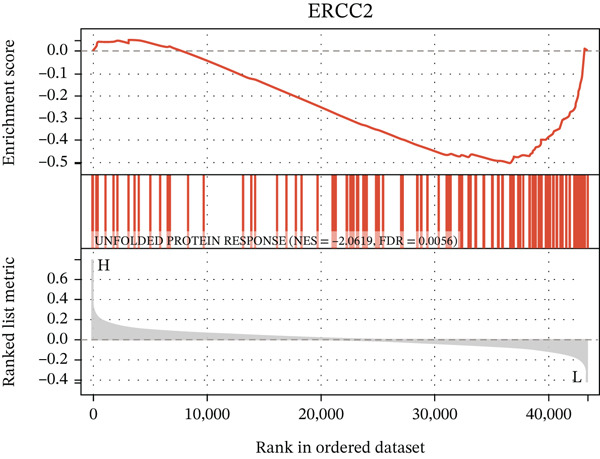
(e)

**Figure:**
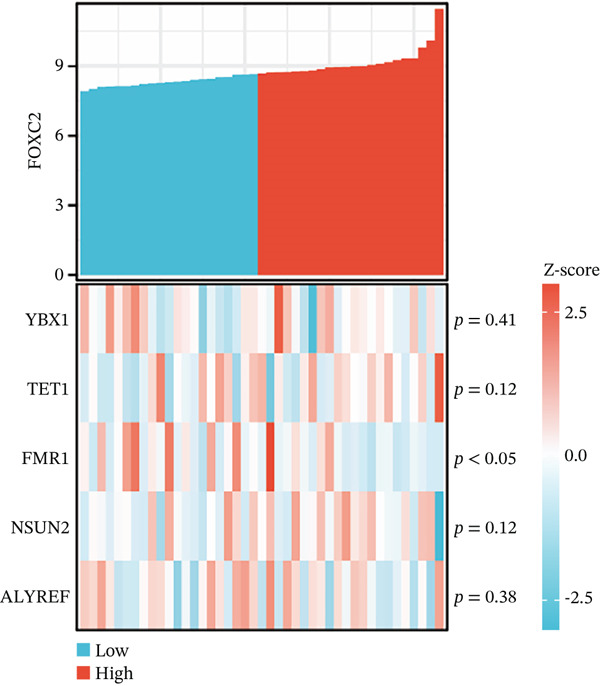
(f)

**Figure:**
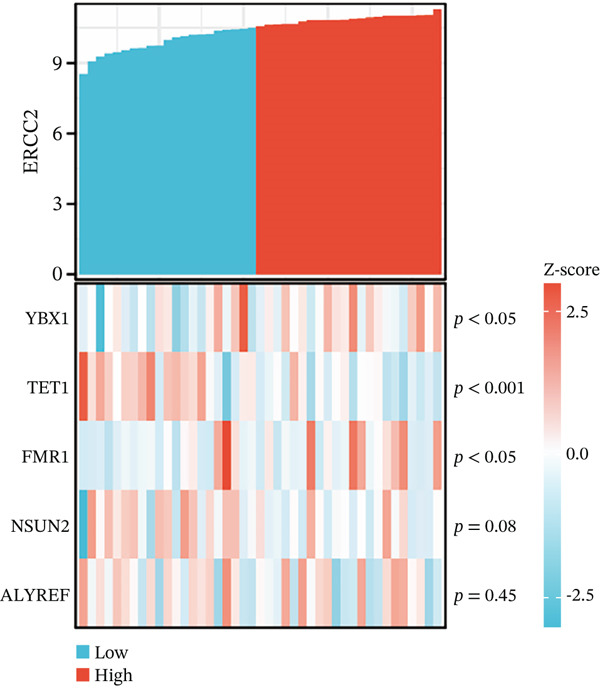
(g)

### 3.4. ERCC2 and FOXC2 Diagnostic Performance Evaluation for NASH Patients

We systematically evaluated the diagnostic performance of ERCC2 and FOXC2 across three datasets: the training set (GSE164760), internal validation set (GSE89632), and independent validation set (GSE63067). The results from all three datasets demonstrate that ERCC2 and FOXC2 are highly expressed in NASH patients, and both genes hold promise as potential diagnostic biomarkers for NASH (Figures 5a, 5b, and 5c).

Figure 5m5C‐associated hub gene diagnostic performance for NASH patients. (a) Comparison of hub gene expression between control and NASH groups, ROC curves, precision–recall curve, net benefit analysis, nomogram plot, and calibration plots of hub genes in training set (GSE164760). (b) Comparison of hub gene expression between control and NASH groups, ROC curves, precision–recall curve, net benefit analysis, nomogram plot, and calibration plots of hub genes in internal set (GSE89632). (c) Comparison of hub gene expression between control and NASH groups, ROC curves, precision–recall curve, net benefit analysis, nomogram plot, and calibration plots of hub genes in Independent validation set (GSE63067).

**Figure:**
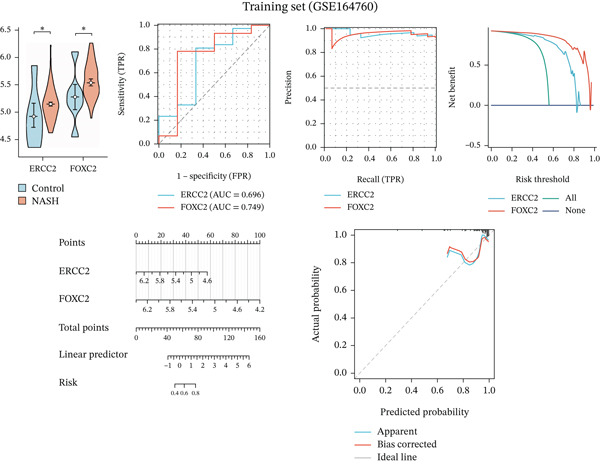
(a)

**Figure:**
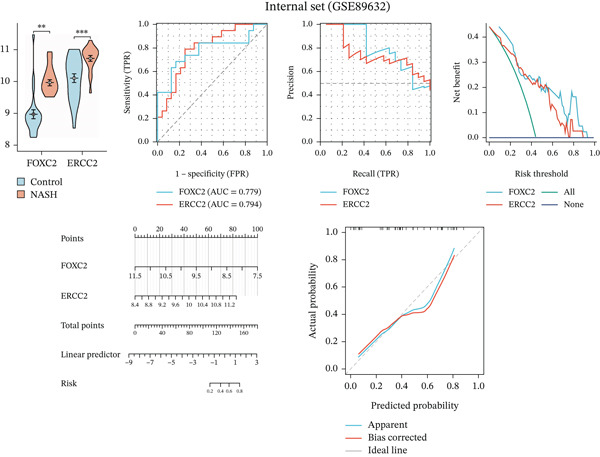
(b)

**Figure:**
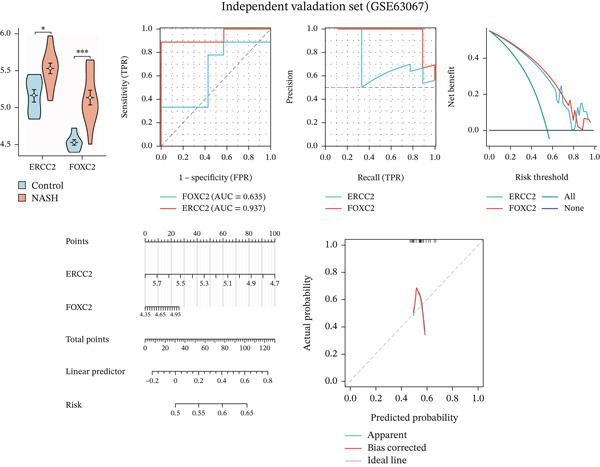
(c)

### 3.5. ERCC2 and FOXC2 Performance Evaluation at Single‐Cell Level of NASH Patients

After pre‐processing of NASH single‐cell transcriptomic profile, we identified 23 cell clusters and nine cell types (Figures S1A, S1B, S1C, S1D, S1E, and S1F and 6a,b). Next, the cell communication and metabolic heterogeneity among nine distinct cell types were also estimated (Figure 6c,d). Expression analysis of ERCC2 and FOXC2 across different cell types indicated significantly higher expression levels in cholangiocytes (Figure 6e). Additionally, pseudotime analysis revealed the differentiation of cholangiocytes, and temporal and spatial ERCC2 and FOXC2 expression patterns were also estimated (Figure 6f,g). Next, we performed AI‐driven KO of ERCC2 and FOXC2 in cholangiocytes and illustrated Top 10 expressed changed molecules (Figure 6h). Significantly, we discovered that after KO of FOXC2 and ERCC2, functions related to RNA polymerase were changed, highlighting their potential role in regulating m5C in cholangiocytes (Figure 6i).

Figure 6The heterogeneity of NASH and FOXC2 with ERCC2 in NASH at single‐cell level. (a) Single‐cell analysis of the distribution of different cell types in NASH single‐cell dataset. (b) Cell proportion of different cell types in NASH single‐cell dataset. (c) Cell talker analysis of different cell types in NASH single‐cell dataset. (d) Metabolic pattern analysis of different cell types in NASH single‐cell dataset. (e) Expression levels of ERCC2 and FOXC2 across various cell types in NASH. (f, g) Pseudotime analysis of ERCC2 and FOXC2 in cholangiocytes. (h, i) Virtual KO of ERCC2 and FOXC2 in cholangiocytes. Single‐gene GSEA of FOXC2 and ERCC2 in HNSCC.

**Figure:**
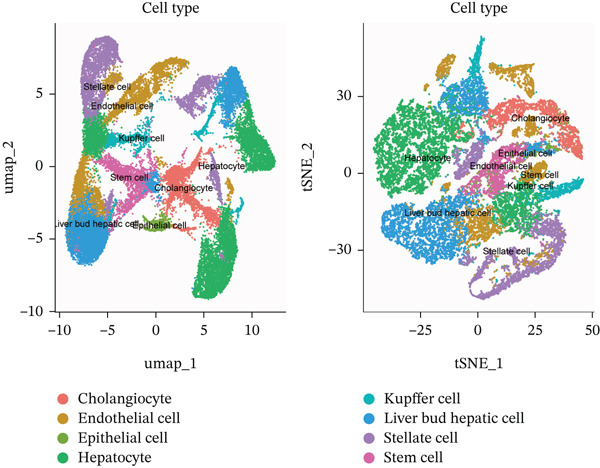
(a)

**Figure:**
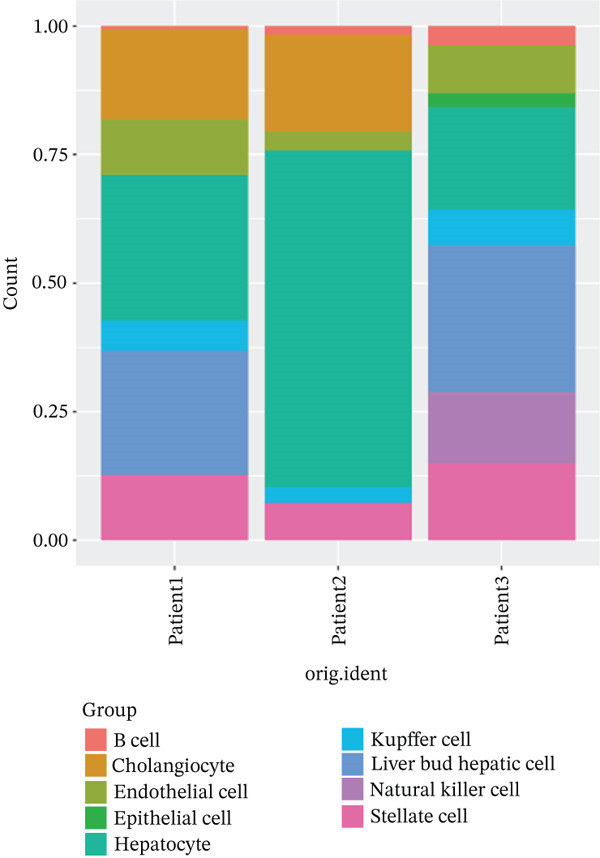
(b)

**Figure:**
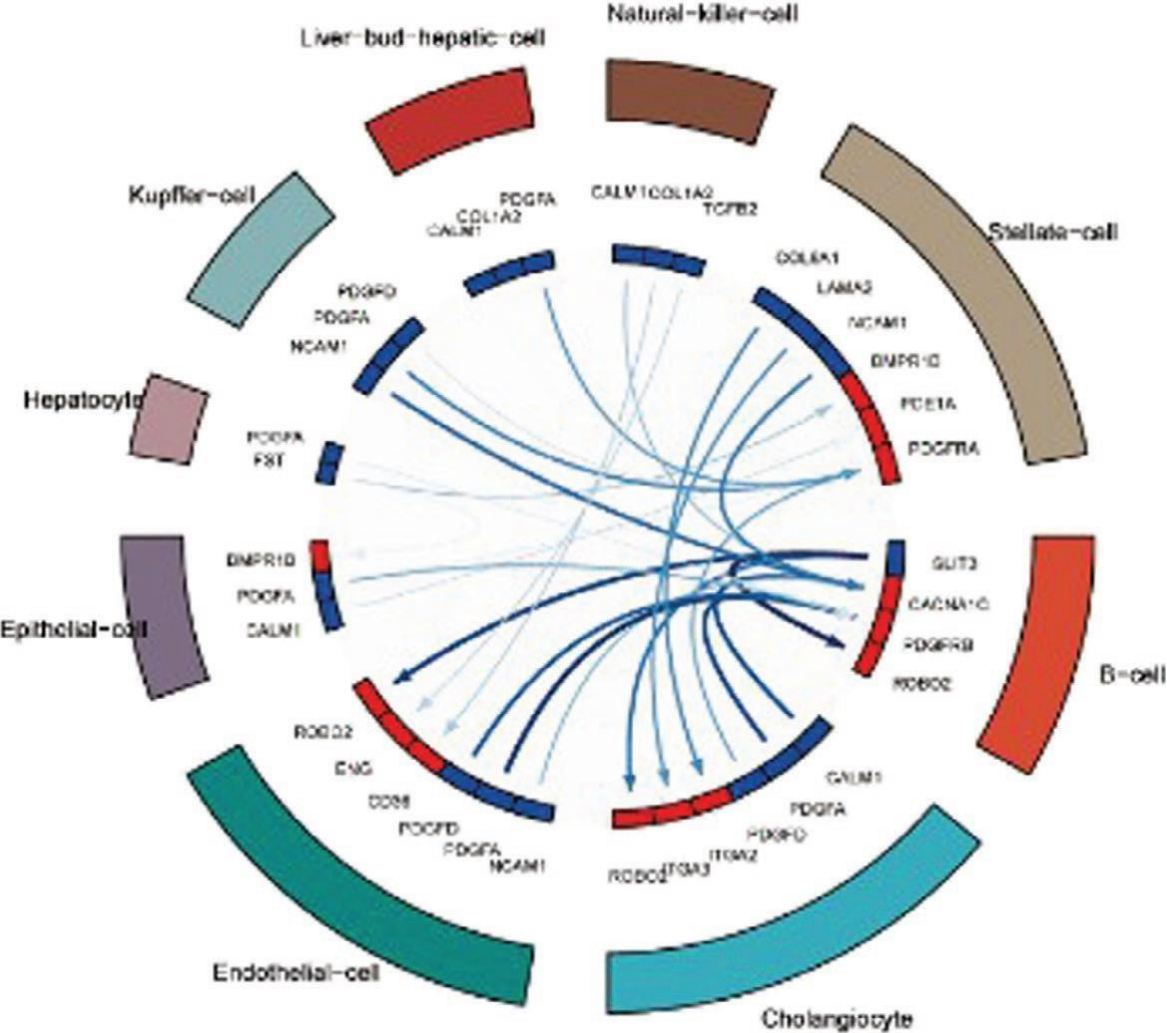
(c)

**Figure:**
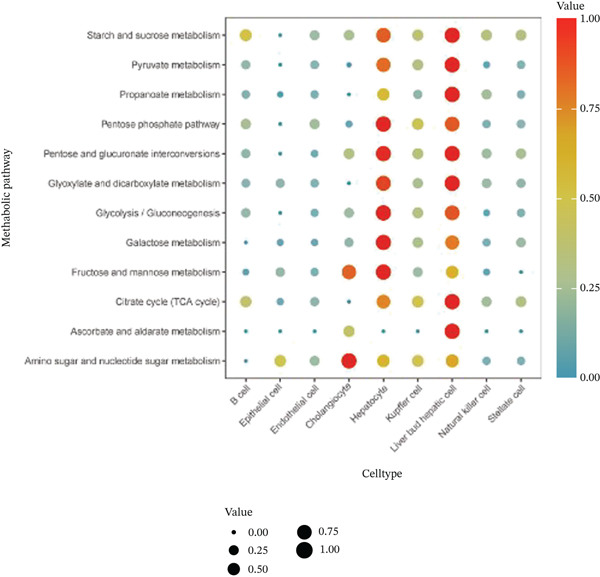
(d)

**Figure:**
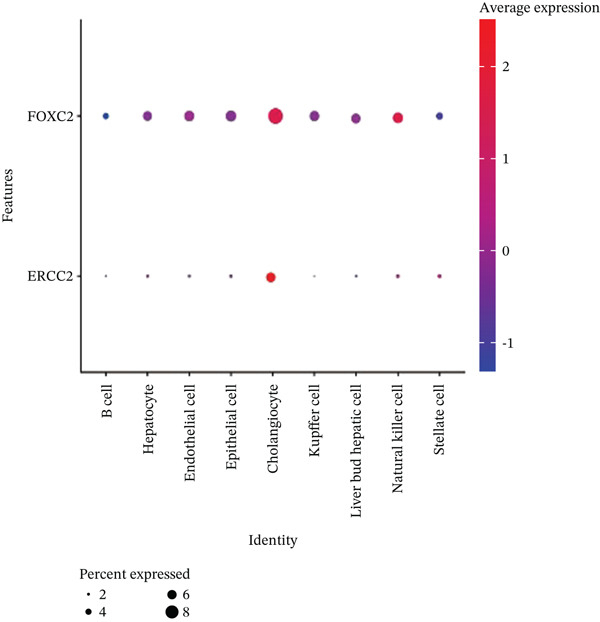
(e)

**Figure:**
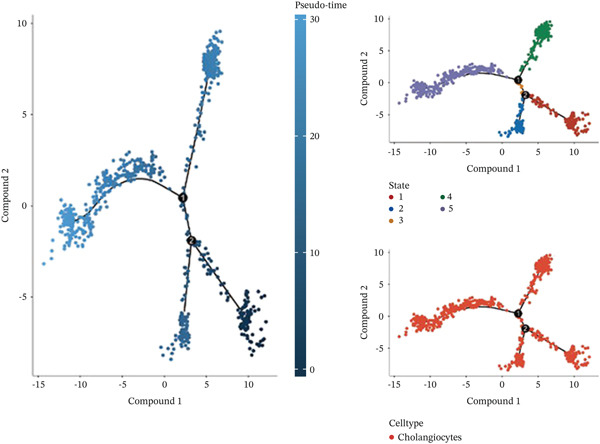
(f)

**Figure:**
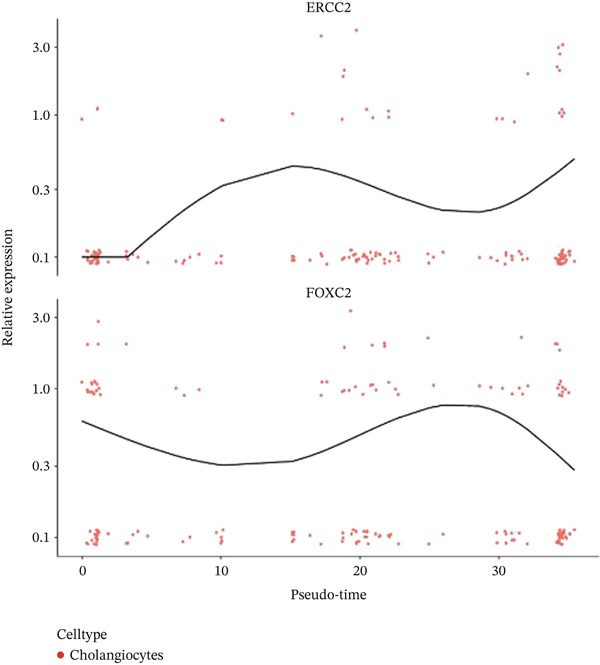
(g)

**Figure:**
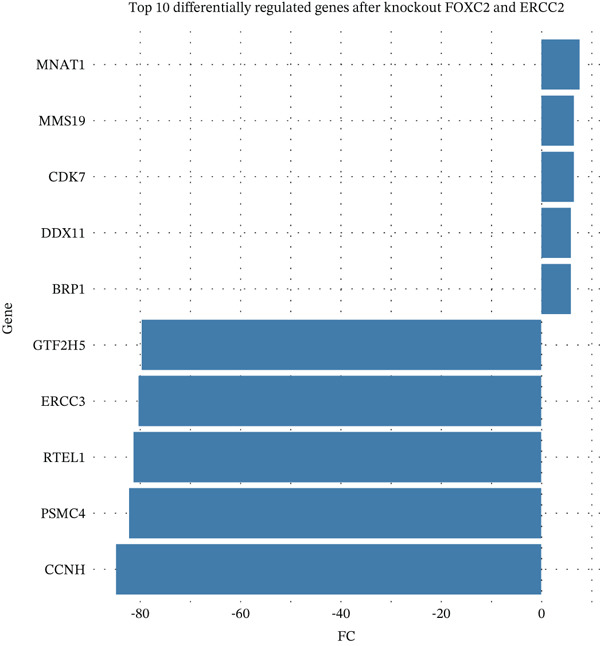
(h)

**Figure:**
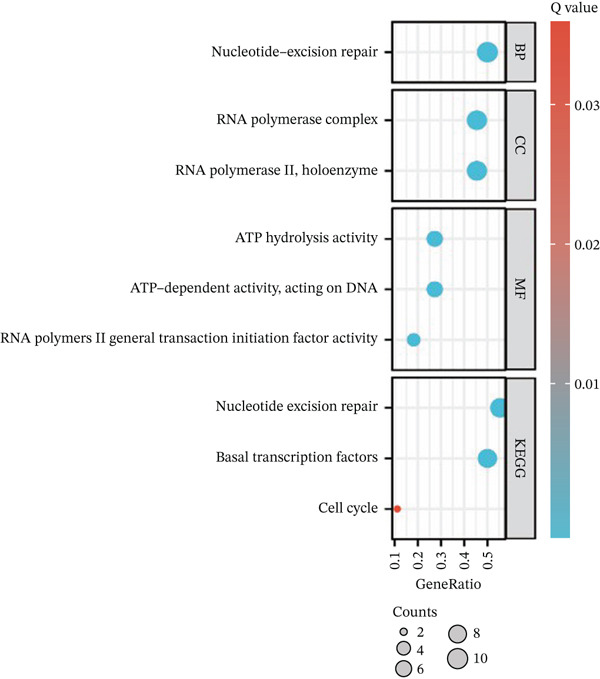
(i)

### 3.6. In Vitro Examination of ERCC2 and FOXC2 Expressions and Targeted Therapeutic Framework Construction

To validate the accuracy of the above results, we performed in vitro analysis to examine the expression of ERCC2 and FOXC2 in a NASH LO‐2 cell model compared to normal LO‐2 cell control. Results indicated that ERCC2 and FOXC2 exhibited markedly elevated levels in both mRNA and protein in the NASH LO‐2 cell model compared to the normal LO‐2 cell control (Figures 7a, 7b, 7c, and 7d). Next, we utilized algorithms in GSE164760 and identified 10 optimal therapeutic agents for the treatment of NASH (Figure 7e). Next, the Top 1 drug (BRD‐K93672499) among the 10 optimal therapeutic agents was selected (Figure 7f). To assess whether ERCC2 and FOXC2 can be considered targets of BRD‐K93672499, we performed molecular docking validation (Figure 7g). Results indicated that there is favorable binding affinity between ERCC2 and FOXC2 and BRD‐K93672499 (Figure 7g).

Figure 7Therapeutic agents enrichment targeting ERCC2 and FOXC2 for treatment of NASH. (a) Relative ERCC2 mRNA levels in the NASH and control groups. (b) Relative ERCC2 protein levels in the NASH and control groups. (c) Relative FOXC2 mRNA levels in the NASH and control groups. (d) Relative FOXC2 protein levels in the NASH and control groups. (e) AI‐driven therapeutic screening for treatment of NASH. (f) 2D illustration of BRD‐K93672499. (g) Molecular docking validation.

**Figure:**
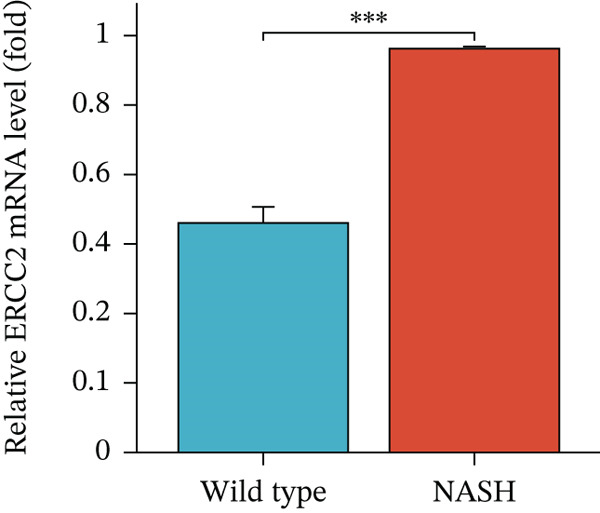
(a)

**Figure:**
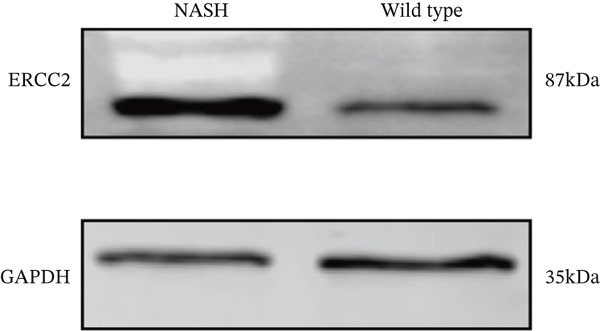
(b)

**Figure:**
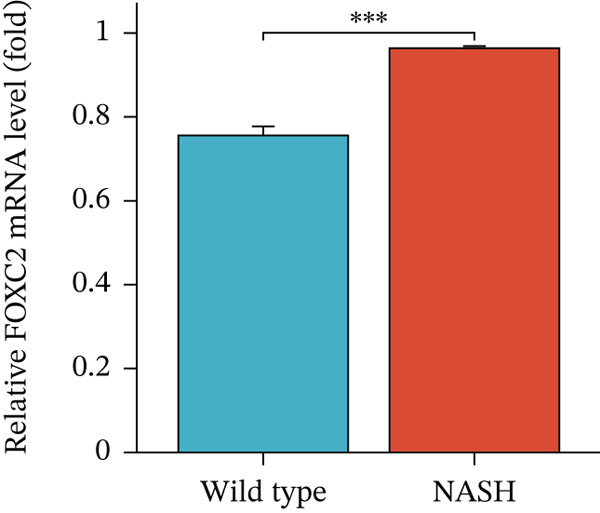
(c)

**Figure:**
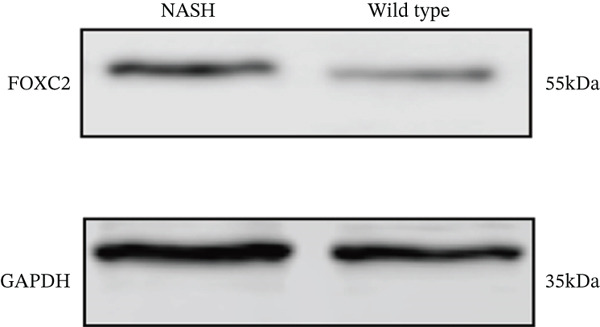
(d)

**Figure:**
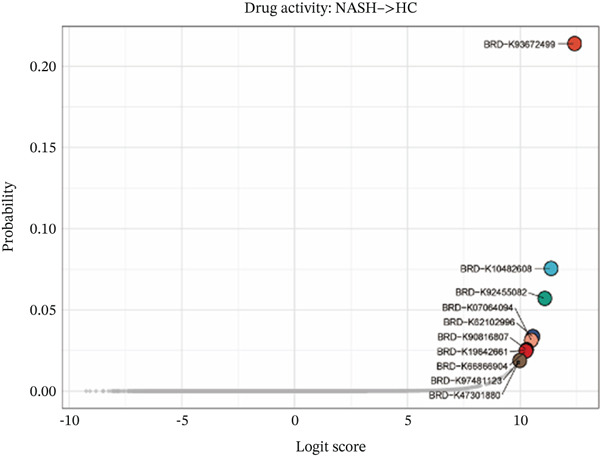
(e)

**Figure:**
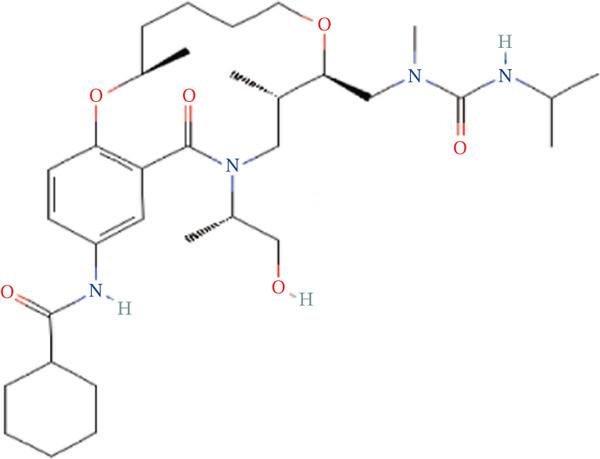
(f)

**Figure:**
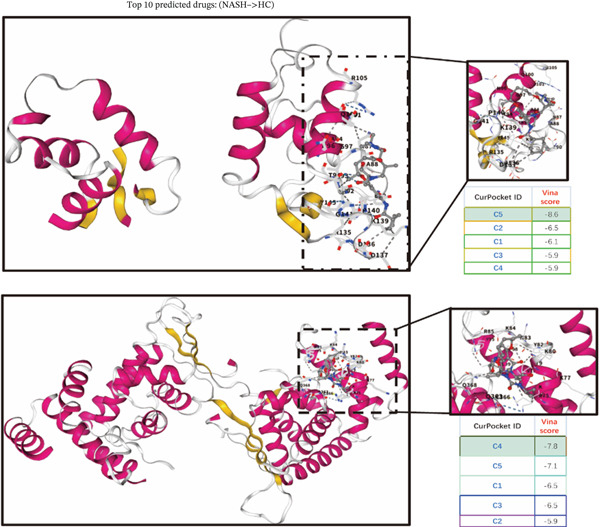
(g)

## 4. Discussion

In this study, we comprehensively explored the involvement of m5C‐related epigenetic modifications in the pathogenesis of NASH by integrating multiomics data with AI. Briefly, we identified novel m5C‐related molecular subgroups and predictive and druggable hub genes (ERCC2 and FOXC2) among NASH patients, which provides additional insights into treatment of NASH.

Mechanistically, aberrant m5C modification serves as a key epigenetic driver linking lipid metabolism, oxidative stress, and inflammation in hepatic disorders. Dysregulated m5C affects RNA stability and translation, influencing pathways such as glycolysis, fatty acid oxidation, and unfolded protein response [25]. ERCC2, a nucleotide–excision repair helicase, maintains genomic and mitochondrial integrity under oxidative and lipotoxic stress, while FOXC2, a Forkhead transcription factor, regulates lipid metabolism, angiogenesis, and epithelial–mesenchymal transition [26, 27]. In the field of m5C, a report indicated that NOP2‐mediated m5C methylation of ERCC2 is associated with hepatocellular carcinoma progression [28]. In addition, m5C also can induce the stabilization of FOXC2 mRNA and increase its expression for promoting gastric cancer cell progression via YBX1 and NSUN2 [29]. However, the m5C role and FOXC2 and ERCC2 m5C mechanisms in NASH pathogenesis have not yet been reported. Significantly, in our study, we first identified the predictive role of m5C for NASH patients and highlighted the FOXC2 and ERCC2 relevance with m5C among NASH patients, which provides additional choice for NASH clinical translation.

## 5. Conclusions

This study identifies ERCC2 and FOXC2 as novel m5C‐related biomarkers and potential therapeutic targets for NASH. Our multiomics approach, combining AI and experimental validation, reveals their diagnostic value and association with key pathological processes for NASH patients. In this study, we first discovered the mechanisms and predictive potentials of m5C for NASH patients, which provides novel prospective in NASH clinical translation. However, ERCC2 and FOXC2 definite molecular m5C‐related mechanisms and BRD‐K93672499 therapeutic effects should be validated in preclinical and clinical studies to enhance robustness.

## Author Contributions

Shuxian Chen and Renquan Duan contributed equally to this work and should be considered co‐first authors.

## Funding

This research did not receive any specific grant from funding agencies in the public, commercial, or not‐for‐profit sectors.

## Disclosure

All the authors read and approved the final manuscript. The work was performed as part of the authors′ employment at their respective institutions.

## Conflicts of Interest

The authors declare no conflicts of interest.

## Supporting information


**Supporting Information** Additional supporting information can be found online in the Supporting Information section.. Figure S1: Preprocessing of single‐cell data. (A) Violin plot showing the distribution of feature counts, RNA levels, and mitochondrial gene percentages across different cell types. (B) Scatter plot illustrating the relationship between RNA counts, feature counts, and mitochondrial gene percentages, with data grouped by patient and replicate. (C) PCA plot displaying the distribution of different cell cycle phases (G1, G2M, and S) in the NASH dataset. (D) *t*‐SNE plot showing the clustering of different cell types in the NASH dataset. (E) UMAP plot visualizing the distribution of cell types in the NASH dataset. (F) Heatmap displaying the expression of key genes across different cell types in the NASH dataset.

## Data Availability

The datasets generated and/or analyzed during the current study are available in the Gene Expression Omnibus (GEO) repository, under accession numbers GSE89632, GSE164760, GSE63067, and GSE189600.
