# Supplementary material for: m5C: Novel Diagnostic and Drug Repurposing Targets for Nonalcoholic Steatohepatitis
Source: Int J Genomics. 2026 Feb 25;2026:4309290. doi: 10.1155/ijog/4309290 (PMC12933632; doi:10.1155/ijog/4309290)
Supplement: Supplementary file 1 — Supporting Information Additional supporting information can be found online in the Supporting Information section.. Figure S1: Preprocessing of single‐cell data. (A) Violin plot showing the distribution of feature counts, RNA levels, and mitochondrial gene percentages across different cell types. (B) Scatter plot illustrating the relationship between RNA counts, feature counts, and mitochondrial gene percentages, with data grouped by patient and replicate. (C) PCA plot displaying the distribution of different cell cycle phases (G1, G2M, and S) in the NASH dataset. (D) t‐SNE plot showing the clustering of different cell types in the NASH dataset. (E) UMAP plot visualizing the distribution of cell types in the NASH dataset. (F) Heatmap displaying the expression of key genes across different cell types in the NASH dataset. [file IJOG-2026-4309290-s001.docx]

**
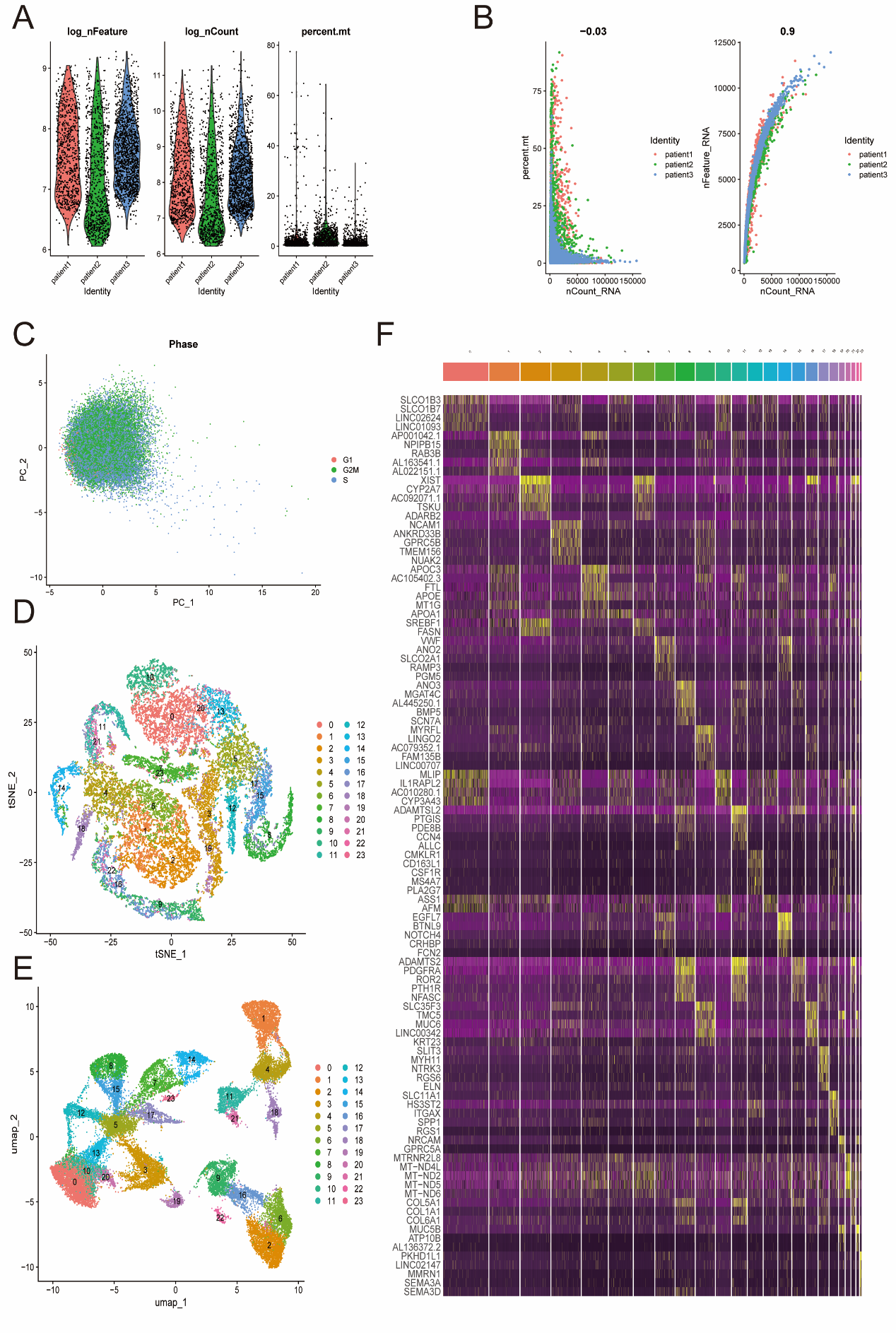
**

**Figure S1:Pre-processing of single-cell data. (A)** Violin plot showing the distribution of feature counts, RNA levels, and mitochondrial gene percentages across different cell types. **(B)** Scatter plot illustrating the relationship between RNA counts, feature counts, and mitochondrial gene percentages, with data grouped by patient and replicate. **(C)** PCA plot displaying the distribution of different cell cycle phases (G1, G2M, S) in the NASH dataset. **(D)** t-SNE plot showing the clustering of different cell types in the NASH dataset. **(E)** UMAP plot visualizing the distribution of cell types in the NASH dataset. **(F)** Heatmap displaying the expression of key genes across different cell types in the NASH dataset.
